# Type of musical soundtrack affects behavior in gambling

**DOI:** 10.1556/JBA.3.2014.006

**Published:** 2014-02-03

**Authors:** RUNE A. MENTZONI, JON CHRISTIAN LABERG, GEIR SCOTT BRUNBORG, HELGE MOLDE, PALLESEN STÅLE

**Affiliations:** ^1^Department of Psychosocial Science, University of Bergen, Bergen, Norway; ^2^Norwegian Institute for Alcohol and Drug Research, Oslo, Norway

**Keywords:** gambling, music, gambling behavior, structural characteristics

## Abstract

*Background and aims:* A long existing notion is that the presence of music might affect gambling behavior. In spite of this, little empirical research on the subject exists. The main aim of the present study was to corroborate and elaborate on the existing findings concerning gambling and music through a laboratory based experiment. *Methods:* A nonclinical sample of 101 undergraduate students (72 females, 29 males) played a computerized gambling task in which either a high-tempo or a low-tempo musical soundtrack was present. Persistence in gambling, reaction time and evaluation of the game comprised the outcome variables. *Results:* Low-tempo music was associated with increased gambling persistence in terms of overall number of bets placed, whereas high-tempo music was associated with intensified gambling in terms of faster reaction time per placed bet. Type of soundtrack was not associated with game evaluation. *Discussion:* Our findings add to the existing knowledge by showing that both low-tempo and high-tempo music can be associated with more risky gambling behavior, the former by increasing gambling persistence and the latter by reducing reaction time for bets placed. *Conclusions:* In sum, the existing studies provide compelling evidence that music can affect various aspects of gambling behavior. These findings may have clinical implications by educating gamblers on the effects of structural mechanisms in gambling on behavior.

In recent years, research on structural mechanisms and significant events in gambling has received increased attention, showing how factors such as note acceptors (Blaszczynski, Sharpe & Walker, 2001; Blaszczynski, Sharpe, Walker, Shannon & Coughlan, 2005), game tempo (Chóliz, 2010; Linnet, Rømer Thomsen, Møller & Callesen, 2010; Mentzoni, Laberg, Brunborg, Molde & Pallesen, 2012), music (Dixon, Trigg & Griffiths, 2007; Spenwyn, Barrett & Griffiths, 2010) and sequential occurrence of wins (Kassinove & Schare, 2001; Mentzoni, Laberg, Brunborg, Molde & Griffiths, 2012; Weatherly, Sauter & King, 2004) affect within-session gambling behavior and cognitions.

For instance, removing note acceptors has been suggested as a harm reducing effort in electronic gambling machines (Blaszczynski et al., 2001), and at least one empirical study has supported this by showing reduced frequency of slot machine gambling and proportion of problem gamblers after the introduction of a nationwide ban on note acceptors (Hansen & Rossow, 2010). With regards to game tempo, it has been demonstrated that at-risk and problem gamblers show more intensive gambling when there is a short bet-to-outcome interval compared to intervals with longer delays between the placed bet and outcome (Chóliz, 2010; Linnet et al., 2010; Mentzoni, Laberg, Brunborg, Molde & Pallesen, 2012). Studies investigating effects of sequential occurrence of wins have reported inconsistent findings. Studies based on retrospective self-report data (Griffiths, 1990a, 1990b, 1991; Johansson & Gotestam, 2003) have suggested a link between the experience of early big wins and later development of problem gambling, but prospective experimental studies have not confirmed this in a consistent way. Two studies failed to show a relationship between early occurring big wins and intensified gambling behavior within the same gambling session (Kassinove & Schare, 2001; Mentzoni, Laberg, Brunborg, Molde & Griffiths, 2012), whereas a third study reported the opposite, that participants experiencing a late occurring big win showed intensified subsequent gambling compared to participant experiencing an early big win (Weatherly et al., 2004).

Studies have shown that music can affect behavior in a broad range of activities. For example, in physical exercise, music has been shown to have beneficial effects both in the pre-task period (i.e. when preparing for exercise; Karageorghis & Priest, 2011a), in the task period (i.e. during exercise) and in the post-task period (Karageorghis & Priest, 2011b). In retail settings, the presence of music has been demonstrated to have a positive effect on patronage, as well as on perceived pleasure (Garlin & Owen, 2006). With regards to cognitive abilities, listening to music has been shown to have beneficial short-term effects such as superior spatial abilities in participants listening to classical music compared to participants in a silent condition and participants who received relaxation instructions (Rauscher, Shaw & Ky, 1993). Long lasting effects have also been reported, for instance from childhood music lessons (Schellenberg, 2005).

The notion that music might influence gambling behavior is not new. In 1912, one author stated that “…no doubt the addition of music to the slot machines is intended to create a passion for throwing away nickels and dimes” (Quinn, 1969, p. 225), although he did go on to note that “it is very seldom that the music charms them back again” (Quinn, 1969, p. 225). However, in spite of this long present notion and the fact that almost every slot machine emits music or sounds, empirical studies of the effects of music on gambling behavior remain disproportionately sparse.

One study, investigating variations in tempo and volume of ambient casino sounds and music present in gambling situations, found that the presence of music helped gamblers to more accurately estimate elapsed time, whereas participants who only heard ambient casino sounds tended to underestimate the duration of play (Noseworthy & Finlay, 2009). Thus, the presence of music could actually help gamblers monitor the extent of their gambling at least in terms of playing time. However, other studies have found that music might lead to more intensive gambling. For instance, one study found that participants watching a video where music replaced ambient casino sounds reported increased at-risk gambling intentions when the video displayed casino environments designed to elicit a playground feeling (i.e. focusing on spaciousness, warm colours, vegetation and moving water; Marmurek, Finlay, Kanetkar & Londerville, 2007). However, the opposite was found when the video displayed casino environments where attention was directed towards gambling equipment specifically. In such environments, music lead to decreased at-risk intentions. In an experiment investigating roulette players in a laboratory setting, it was found that faster betting occurred while high-tempo music was played, whereas bet size and overall amount spent was not influenced (Dixon et al., 2007). A more recent study, also investigating roulette players, reported similar results: Increased music tempo lead to faster betting, but not increased risk-taking (Spenwyn et al., 2010). Thus, the studies so far seem to indicate that certain aspects of music (such as tempo) are likely to affect gambling intensity in terms of betting speed,

The finding that high-tempo music is associated with intensified gambling runs partially counter to findings from reports on consumer behavior in different retail settings. For instance, restaurant customers have been found to spend more time and money when low-tempo rather than high-tempo music is played (Caldwell & Hibbert, 1999; North, Shilcock & Hargreaves, 2003). However, the studies from gambling situations have focused on individual bet-sizes rather than total spending.

Our aim with the present study was to corroborate and elaborate on the existing findings concerning gambling and music. Since previous laboratory based studies have investigated roulette playing, we employed a different task in order to investigate if the findings would generalize to other forms of gambling. Secondly, while previous studies used music as background sound we embedded the music in the gambling task so that the music would be experienced as part of the task, rather than as a background factor. Thirdly, in order to investigate if music tempo could influence total time spent gambling, and thereby total money spent, we assessed number of bets placed rather than mean bet size as an outcome variable. Finally, previous studies have linked tempo of music with specific gambling behavior (i.e. speed of placing bets). In order to assess if music is associated with the overall valence of the gambling experience, we asked participants to indicate the degree to which they enjoyed the gambling task. Based on previous findings, we expected that tempo of music would affect betting speed in terms of faster reaction time when listening to faster music. Further, we expected that the total time spent gambling would be longer (i.e. more placed bets) when low-tempo music was played as opposed to when high-tempo music was played. The measure of game evaluation was exploratory, and no hypothesis was made regarding this outcome variable.

## METHODS

### Participants

A total of 101 participants were recruited, 72 females and 29 males. Mean age was 21.0 years (*SD* = 2.26), with a range of 18 to 29. All participants were undergraduate psychology students from the University of Bergen. Prior to the experiment, participants consented to take part in a computerized gambling task in which a start-up credit would be provided for gambling, and where any wins obtained during the task would be paid out in cash upon completion. No specific details were given about the content of the gambling task, or about the chances of winning.

### Apparatus and measures

*Gambling simulation.* The gambling task, “*SuperJack*”, was programmed using E-prime 2. On each trial, four playing cards were displayed, picture sides facing down. The task was to select one of the cards by pressing one of four marked keys on a standard QWERTY keyboard. Each trial (bet) cost NOK 3 ($0.50), and a start-up credit of NOK 50 ($9) was provided. Before gambling started, the participants were verbally informed of the following: If the selected card turned out to be a Queen, King or Ace a small win of NOK 3 ($0.50) would be obtained. A Jack of any suit would yield a win of NOK 20 ($3.50). A Joker would win NOK 100 ($18). A *SuperJack* (special card) would win NOK 250 ($44.50), and two consecutive SuperJacks would win NOK 850 ($151). Any other card resulted in no win on that trial. This information was also available on a sheet placed within eyesight during the gambling task.

*South Oaks Gambling Screen Revised.* The South Oaks Gambling Scale Revised (SOGS-R; Lesieur & Blume, 1993) was administered in order to detect the presence of any pre-existent gambling problems among participants. SOGS-R indicates a person’s level of problem gambling, with a maximum score of 20. According to Lesieur and Blume (1993) a score of 0 indicates “no problems with gambling”, a score of 1–4 indicates “some problems with gambling”, and a score of 5 or more indicates “probable pathological gambler”. The majority of participants (66) had no problems with gambling (SOGS-R = 0), 34 participants had some gambling problems (SOGS-R scores of 1–4) and one participant scored at the cut-off for probable pathological gambling (SOGS-R = 5).

*The Bergen Evaluation of Games Scale.* An 8-item scale (Bergen Evaluation of Games Scale; BEGS) was developed in order to measure the degree to which participants found the gambling enjoyable. Upon completion of the gambling session, participants rated how much they agreed to statements like “All in all, I enjoyed playing the game” on a 7-point Likert-scale. See Appendix for a complete English translation of this scale. Cronbach’s alpha for BEGS was .95 in the present study.

*Reaction time (RT).* RT was measured in milliseconds, recorded from the point where the fourth card occurred on-screen, to the participant’s response (i.e. choice of card) on each placed bet. Median RT from the 20 trials following trial number 5 was used, allowing five trials of practice.

### Procedure

The experiment was conducted in individual testing booths within a purpose built laboratory at the University of Bergen. Each booth contained a desktop computer and an office chair. The testing booths were sound attenuated, and sound effects and musical soundtrack was presented via headphones. Sound effects were presented for the following in-game events: Dealing of cards, small win, medium win, large win and loss.

Participants were informed that they would be asked to partake in a gambling session with a start-up credit of NOK 50. Upon completion of the experiment, any wins obtained would be paid out in cash. Participants could play for as long as they liked, or until they ran out of money. Unknown to the participants, outcomes were pre-programmed, and the participants’ selections had no influence on winnings. Participants would run out of money after 86 trials (if they had not quit playing earlier), during which they would have experienced nine small wins (NOK 3), four medium wins (NOK 20) and one large win (NOK 100). The NOK 250 and NOK 850 wins never occurred.

Two different musical soundtracks were used; half of the participants played with a slow-paced jazz music sound-track, the other half with a fast-paced pop melody. Both soundtracks were instrumental MIDI tracks played through the computer’s onboard sound card synthesizer.

SOGS-R was completed prior to testing. BEGS was completed after each gambling block.

### Ethics

The study was carried out in accordance with the Declaration of Helsinki. The Regional Committee for Medical Research Ethics, Health Region West, Norway approved the study. All participants were informed about the study, and all provided informed consent.

## RESULTS

### Correlations

Bivariate correlations between the three outcome variables (bets placed, RT and game evaluation) and SOGS-R score are shown in Table 1. Game evaluation was significantly and positively correlated with RT. No other significant correlations were found.

**Table 1.** Means, standard deviations and correlations between study variables (*N* = 101)

Variables	Mean	*SD*	1	2	3	4
1. Games played	72.10	24.48	–			
2. Reaction time (ms)	621	439	–0.17	–		
3. Game evaluation	3.69	1.21	0.02	0.25^*^	–	
4. SOGS-R score^a^	0.54	0.92	0.17	–0.02	–0.07	–

^a^ Coefficients are Spearman correlations;

^*^ *p* < .05.

### Bets placed

An independent samples *t*-test was conducted, comparing mean number of bets placed in the two conditions of musical soundtrack. A significant difference was found, indicating that participants in the slow music tempo group placed more bets compared to participants in the fast tempo group, *M* = 77.3, *SD* = 19.2 and *M* = 66.9, *SD* = 27.8, respectively, *t*(1,99) = 2.20, *p* = .03. This was in line with our hypothesis.

### Reaction time

A univariate ANCOVA was conducted in which RT was entered as the dependent variable, and Soundtrack (fast or slow) as the fixed factor. Game evaluation was significantly and positively correlated with RT (see Table 1), and was therefore entered as a covariate. Two participants displayed a response bias in which the same card was chosen on all trials, which is likely to have affected their RT. Consequently, these cases were dropped from the analysis. The ANCOVA revealed a significant main effect of game evaluation on RT, *F*(1, 93) = 6.55, *p* = .01, and a significant main effect of soundtrack on RT, *F*(1, 93) = 6.10, *p* = .02. Participants with the up-tempo soundtrack displayed faster RT compared to participants with the down-tempo soundtrack, *M* = 519, *SE* = 57.2 and *M* = 722, *SE* = 58.5, respectively. Thus, the prediction that musical tempo would influence RT was supported.

### Game evaluation

In order to investigate if musical tempo in the gambling task was associated with the participants’ evaluation of the game, an independent samples *t*-test was conducted. The analysis revealed that there was no difference in game evaluation in the high-tempo compared to the low-tempo condition, *M* = 3.8, *SD* = 1.2 and *M* = 3.6, *SD* = 1.2, respectively.

## DISCUSSION

Participants who listened to a low-tempo soundtrack while gambling displayed prolonged gambling behavior, by placing more bets than did participants listening to a high-tempo soundtrack while gambling. Thus, our predictions were supported. It could be that the low-tempo music had a relaxing effect on participants, and that this state of relaxation made continued gambling more likely even though no effect on self-reported game evaluation was evident.

Our hypothesis that high-tempo music would be associated with faster reaction times when placing the bets was also supported. This corroborates previous findings (Dixon et al., 2007; Spenwyn et al., 2010), which in sum strongly suggest that type of music exerts an influence on certain aspects of gambling behavior. A novel aspect of our study was the inclusion of a measure of participants’ game evaluation. Interestingly, type of soundtrack did not influence these evaluations, indicating that the observed effect of sound-track on reaction time is most likely unrelated to the perceived valence of the gambling experience.

Our findings add to the existing knowledge by showing that both low-tempo and high-tempo music can lead to more risky gambling behavior, the former by prolonging gambling and the latter by reducing reaction time for bets placed. Furthermore, our findings indicate that the findings are valid for different types of gambling games, by replicating effects found with roulette playing (Dixon et al., 2007; Spenwyn et al., 2010) in an electronic card game.

The findings we present have clinical relevance by demonstrating that gamblers are affected by type of music in a gambling situation and that this effect is independent of how gamblers evaluated the overall gambling situation. This information can be used to educate gamblers about subtle nuances in the behavior they engage in, and to encourage them to employ a self-monitoring approach reflecting upon contextual effects on their own behavior.

To date, experimental studies on the effects of music in gambling have relied on samples consisting of recreational gamblers. Consequently, it cannot be ruled out that music might have a different effect on pathological gamblers. Thus, future studies should include pathological gamblers in order to build on the existing knowledge. Further, studies so far have been conducted in laboratory settings, and it remains unknown how this has affected the findings. Data from naturalistic settings would be a highly welcomed addition to the field.

In conclusion, low-tempo and high-tempo music affects different aspects of gambling behavior and both types may lead to more risky gambling through different mechanisms. This information could be used to inform gamblers interested in self-monitoring their behavior.

## APPENDIX

### THE BERGEN EVALUATION OF GAMES SCALE (BEGS)

Below you will find a list of claims regarding the game you just played. Please read every claim carefully, and rate the degree to which you agree or disagree by circling the appropriate number (1–7).

1. All in all, I enjoyed playing the game

Completely disagree 1 2 3 4 5 6 7 Completely agree

2. The game was a positive experience for me

Completely disagree 1 2 3 4 5 6 7 Completely agree

3. The speed of the game suited me fine

Completely disagree 1 2 3 4 5 6 7 Completely agree

4. I would recommend the game to a friend

Completely disagree 1 2 3 4 5 6 7 Completely agree

5. If given the opportunity, I would like to play the game again

Completely disagree 1 2 3 4 5 6 7 Completely agree

6. The game did not suit me

Completely disagree 1 2 3 4 5 6 7 Completely agree

7. I was quickly bored by the game

Completely disagree 1 2 3 4 5 6 7 Completely agree

8. I was engaged by the game

Completely disagree

1 2 3 4 5 6 7 Completely agree

Items 6 and 7 are reverse scored.
